# Reactivation of Occult Hepatitis B Virus Infection During Long-Term Entecavir Antiviral Therapy

**DOI:** 10.3389/fmicb.2022.865124

**Published:** 2022-03-10

**Authors:** Chunyan Yuan, Jing Peng, Renxiang Xia, Jian He, Tianji Qiu, Yunqing Yao

**Affiliations:** Department of Infectious Diseases, The First Affiliated Hospital of Chongqing Medical University, Chongqing, China

**Keywords:** occult hepatitis B virus infection, antiviral therapy, S gene mutation, pre-C gene mutation, drug-resistant, entecavir, reactivation

## Abstract

Up to now, it has not been clear whether occult hepatitis B virus (HBV) infection (OBI) can be treated with antiviral therapy whether OBI can develop drug resistance gene mutation or not. We report a middle-aged female patient with OBI who showed HBV reactivation (HBVr) during more than 3 years of intermittent entecavir (ETV) antiviral therapy: seropositive HBV surface antigen (HBsAg), increased e antigen (HBeAg), and repeatedly elevated serum HBV DNA. Genotype analysis showed that the patient was infected with HBV type B. Genetic sequencing of HBV showed the mutants of S143T, D144G, and G145R in the S gene region, and the mutant of site 1896 in the pre-Core region coexisted with the wild type (G1896A/G). No mutation was found in other HBV gene segments. Drug resistance gene analysis found RtL229W mutant, resistant to lamivudine but sensitive to ETV and other nucleoside analogs. This case of OBI provides us with the following clinical experiences: Firstly, it is necessary to detect HBV genotype, mutation, and drug-resistant genes at the initial diagnosis, which can be helpful for reasonable treatment. Secondly, identifying the risk factors and mechanisms associated with HBVr could help quantify the risk of HBVr and manage the clinical consequences. Thirdly, the OBI patients with hepatitis B e antigen-positive, HBV DNA > 1 × 10^3^ IU/ml should be recommended regular and continuous antiviral therapy as soon as possible to prevent the occurrence of hepatocirrhosis and hepatocellular carcinoma (HCC).

## Introduction

Occult hepatitis B virus (HBV) infection (OBI) is defined as surface antigen (HBsAg) seronegative, core antibody (HBcAb) seropositive, and HBV DNA positive in serum or liver (de Almeida and de Paula, 2021). OBI may result in HBV reactivation (HBVr), acute exacerbations, cirrhosis, and hepatocellular carcinoma (HCC; Mak et al., 2020). For OBI patients or HBV exposure (HBsAg-negative but HBcAb-positive; Pattullo, 2015), according to the recent American Association for the Study of Liver Diseases (AASLD) recommendation guideline, HBVr could be defined as (1) HBV DNA is detectable; or (2) reverse HBsAg seroconversion occurs (reappearance of HBsAg; Terrault et al., 2018; Onorato et al., 2021).

The occurrence of HBVr largely depends on the primary disease requiring chemotherapy or immunosuppressive therapy, host immunity, underlying disease, and the immunosuppressive agents used. The main risk factors associated with HBVr could be divided into three categories: (1) host factors (such as male sex, older age, presence of liver cirrhosis, and type of underlying diseases); (2) virological factors (the extent of HBV replication is most important); and (3) type of immunosuppressive regimen. The HBVr rate ranges from 8.9 to 41.5% in occult HBV infection patients receiving rituximab-containing chemotherapy in different studies using the definitions of HBsAg seroreversion or detectable HBV DNA (Shih and Chen, 2021).

Here, we report a unique case of OBI who developed HBV reactivation during the long term of antiviral therapy but without chemotherapy or immunosuppressive therapy.

## Medical Condition at Initial Diagnosis

In April 2018, a 55-year-old woman was diagnosed with occult HBV infection during the medical examination. She had no symptoms and signs of liver disease. She denied the history of any liver disease, hepatitis virus infection, anti-HBV treatment, tumor, chemotherapy, biological therapy or immunosuppressive therapy, blood transfusion, diabetes, and tuberculosis. She denied drinking or smoking. She submitted that her mother and a brother had died of liver cancer but had no clear family history of chronic hepatitis B. Her husband and children had no HBV infection.

The laboratory examination results showed that blood routine tests, urine routine tests, liver function, kidney function, and alpha-fetal protein (AFP) were normal.

Hepatitis B virus serological testing showed that HBsAg was negative (less than 0.05 IU/ml), surface antibody (HBsAb) was positive (53.69 mIU/ml, more than ULT 10 mIU/ml), e antigen (HBeAg) was positive (45.42 S/Co, more than 1.0 S/Co), e antibody (HBeAb) was negative (0, less than 1.0 S/Co), and HBcAb was positive (9.37 S/Co, more than 1.0 S/Co. Details shown in Table 1).

**Table 1:** The serological and molecular characterization results during intermittent antiviral therapy from April 2018 to August 2021.

Date	HBsAg (IU/ml)	HBsAb (mIU/ml)	HBeAg (S/Co)	HBeAb (S/Co)	HBcAb (S/Co)	HBV DNA (IU/ml)
April 2018	0	53.69	45.42	0	9.37	2.32 × 10^3^
May 2018	0	63.50	44.60	0	9.41	<100
November 2018	0	38.50	26.93	0	7.66	<100
July 2019	0	66.76	36.76	0	9.62	2.79 × 10^3^
October 2019	0	36.51	29.79	0	8.73	<100
September 2020	0.10	22.08	27.71	0	7.74	<100
August 2021	0.45	0	66.50	0	8.09	5.93 × 10^3^

Serological tests showed that hepatitis A, C, D, and E virus markers were negative.

Real-Time PCR-Fluorescence Quantitation showed that the serum load of HBV DNA was 2.32 × 10^3^ IU/ml. HBV Genotype analysis showed that type B.

Abdominal ultrasound revealed mild fatty liver but no fibrosis, cirrhosis, or HCC. Liver Stiffness Measurement (LSM) was 5.5 kPA (range of normal value was 4.0–7.0 kPA).

## The First Stage of Antiviral Therapy and Follow-Up

After the initial diagnosis of OBI, the patient was recommended oral entecavir (ETV) therapy (single dose 50 mg daily) and long-term outpatient follow-up.

In May 2018, 4 weeks after initiation of ETV treatment, follow-up tests showed that a load of serum HBV DNA dropped below 100 IU/ml (Minimum detection value line). There were no significant changes in other serum markers of HBV except HBsAb was about 10 mIU/ml more than before (63.50 mIU/ml. Details shown in Table 1). There were no significant drug-related adverse events.

In November 2018, 30 weeks after ETV treatment, follow-up tests showed that serum HBV DNA was still less than 100 IU/ml, HBeAg was much less than that of initial diagnosis (26.93 S/Co. Details shown in Table 1). Interestingly, HBsAb also decreased significantly from last time (38.50 mIU/ml. Details are shown in Table 1).

The patient had no symptoms and signs of liver disease.

The laboratory examination results showed that blood routine tests, urine routine tests, liver function, kidney function, and AFP were normal.

Abdominal ultrasound revealed no fatty liver, fibrosis, cirrhosis, or HCC. The result of LSM was normal.

There were no significant drug-related adverse events.

The patient was advised to continue ETV antiviral therapy.

## The First Reactivation of HBV and Drug Resistance Genetic Testing

On a subsequent follow-up visit (July 2019), test results showed that the serum load of HBV rebounded to 2.79 × 10^3^ IU/ml (Details shown in Table 1). Interestingly, serum levels of HBsAb, HBeAg, and HBcAb were higher than those in November 2018 (Details shown in Table 1). Meanwhile, serum HBsAg was still negative, and there was no other abnormal detection. The patient had no symptoms and signs of liver disease.

Further examination of the patient’s medical history revealed that the patient had spontaneously stopped taking ETV for 6 months.

In addition, drug resistance gene examination of HBV showed that RtL229W mutant, resistant to lamivudine but sensitive to ETV and other nucleoside analogs (Details shown in Table 2).

**Table 2:** The results of drug resistance gene examination of hepatitis B virus (HBV; July 2019).

Drug	Genetic locus			Potential phenotype	Comprehensive phenotype	
lamivudine (LAM)	major: rtM204I	rtM204V	rtM204S	S	U	
	minor: rtL80I	rtL80V	rtV173L	S		
	rtL180M	rtA181T	rtA181V			
	undetermined: rtV207I	rtV207L	rtV207M	U		
	rtS213T	rtL229F	rtL229G			
	rtL229M	rtL229V	rtL229W		S	
tibivudine (LdT)	major: rtM204I	rtM204V	rtM204S	S		
	minor: rtV173L	rtL180M		S		
emtricitabine (FTC)	major: rtM204I	rtM204V	rtM204S	S	S	
	minor: rtV173L	rtL180M		S		
adefovir (ADV)	major: rtA181S	rtA181T	rtA181V	S	S	
	rtN236T	rtN236V				
	undetermined: rtV84M	rtS85A	rtS85T	S		
	rtV214A	rtQ215H	rtQ215P			
	rtQ215S	rtI233V	rtP237H			
	rtN/H238D	rtN/H238S	rtN/H238T			
tenofovir (TDF)	major: rtA194T	rtA194M		S	S	
	minor: rtN236T	rtN236V		S		
entecavir (ETV)	major: rtT184A	rtT184C	rtT184F	S	S	
	rtT184G	rtT184I	rtT184L	
	rtT184M	rtT184S	rtS202C	
	rtS202G	rtS202I	rtM250I	
	rtM250L	rtM250V		S
	minor: rtI169T	rtV173L	rtL180M
	rtM204I	rtM204V	rtM204S

Subsequently, this patient was recommended to take ETV antiviral therapy again.

## The Second Stage of Antiviral Therapy and Follow-Up

In October 2019, 3 months of ETV oral treatment, follow-up test results showed that the patient’s serum load of HBV decreased to less than 100 IU/ml, serum level of HBsAb, HBeAg, and HBcAb also decreased to than those in July 2019 (Details shown in Table 1). Meanwhile, serum HBsAg was still negative, and there was no other abnormal detection. The patient had no symptoms and signs of liver disease. There were no significant drug-related adverse events.

The patient was encouraged to continue ETV antiviral therapy.

## The Second Reactivation of HBV

In September 2020, 14 months of uninterrupted ETV oral treatment, follow-up test results showed that the patient’s serum HBsAg became positive (0.10 IU/ml, more than ULT 0.05 IU/ml. Details shown in Table 1), while the serum HBV DNA was undetectable, serum level of HBsAb, HBeAg, and HBcAb further decreased to less than those in October 2019 (all were positive. Details shown in Table 1).

There was no other abnormal detection, no symptoms, and no signs of liver disease. There were no significant drug-related adverse events.

The patient was encouraged to continue ETV antiviral therapy.

## The Third Reactivation of HBV and Genetic Variation Detection

Unfortunately, in August 2021, the patient spontaneously stopped ETV oral therapy for 6 months before seeing a physician. Follow-up test results showed that the patient’s serum HBsAg increased to 0.45 IU/ml, much higher than that of September 2020 (positive, ULT 0.05 IU/ml), the serum load of HBV DNA also increased to 5.93 × 10^3^ IU/ml from negative, serum levels of HBeAg and HBcAb were higher than those in September 2020, while serum HBsAb was negative (Details shown in Table 1).

The patient denied any symptoms and signs of liver disease and other diseases. There was no other abnormal laboratory detection. There were no significant drug-related adverse events.

Abdominal ultrasound revealed no fatty liver, fibrosis, cirrhosis, or HCC. The result of LSM was normal.

Mutation analysis of this patient’s HBV showed there were three mutants (S143T, D144G, and G145R) in the S gene region, and the mutant of site 1896 in the pre-Core region coexisted with the wild type (G1896A/G), but no mutation in the pre-S1, pre-S2, and BCP gene regions (Details shown in Table 3).

**Table 3:** The results of mutation analysis of HBV (August 2021).

Gene site		Wild type/mutant
pre-C	1862	Wild type (G1862)
	1896	mutant coexists with the wild type (G1896A/G)
	1899	Wild type (G1899)
BCP	1762	Wild type (A1762)
	1764	Wild type (G1764)
pre-S1 Deletion mutation		No deletion mutations were detected
pre-S2 Promoter mutation		Wild type promoter (ATG)
pre-S2 Deletion mutation		No deletion mutations were detected
S	118	Wild type (T118)
	120	Wild type (P120)
	126	Wild type (T/I126)
	127	Wild type (P127)
	129	Wild type (Q129)
	131	Wild type (T131)
	133	Wild type (M133)
	143	mutant type (S143T)
	144	mutant type (D144G)
	145	mutant type (G145R)

In summary, due to the S gene mutation and drug resistance gene, the patient had become a chronic HBV infection from OBI.

## Discussion

The case presented here represents a one-of-a-kind serological profile of HBV infection. Negative HBeAg is a common finding in OBI patients. OBI with positive HBeAg has been described but uncommon (Zhou et al., 2009; Han et al., 2015; Raimondo et al., 2019). Because HBeAg is associated with a high level of HBV replication, we can speculate that an escape mutant appeared at the time of diagnosis while the wild-type virus was still present in the patient. Patients are infected with “S-escape” mutations, which result in modified HBsAg that can evade standard assays. Unfortunately, we could not complete the gene sequencing from 2018 to 2020. The S gene sequencing in August 2021 revealed that mutations were detected at three amino acid sites (S143T, D144G, and G145R), while HBsAg was positive, which could be due to wild-type accounting for a large proportion at the time. A high level of HBV DNA is linked to e-antigen status and virus mutation. Although HBeAg is not part of the virus particle and is not required for infectivity or viral replication, its presence in the bloodstream correlates with high levels of viral replication in hepatic tissue. Furthermore, OBI infected with “S-escape” mutations has a higher viral load. As a result, the patient with negative HBsAg but positive HBeAg may represent a distinct type of OBI that warrants further investigation.

The case demonstrates the efficacy of antiviral therapy, as no study has previously reported on the antiviral response in patients with OBI. The patient requires antiviral therapy for the following reasons. In individuals with chronic hepatitis B, the level of HBV DNA is a well-described risk factor for progressive liver disease and a marker of viral replication and antiviral treatment efficacy. Furthermore, the patient’s family had a history of liver cancer. After antiviral therapy, the HBV load was undetectable, and the titer of e antigen gradually decreased, indicating that antiviral therapy was feasible for this patient. The patient had no hepatitis symptoms or signs during treatment, and his alanine aminotransferase level was normal. Further research is needed to determine whether these indicators, such as serum HBV DNA, e-antigen status, and virus mutation, can be used as an antiviral therapeutic indicator in OBI.

After HBV infection, the virus genome remains in host hepatocytes and can reactivate. Common triggers include inappropriate antiviral drug withdrawal and the use of immunosuppressive therapies. The reactivation of HBV in this patient could be attributed to the untimely discontinuation of antiviral medications. In this case, drug withdrawal occurred twice. The first occurred in July 2019, resulting in a resurgence of the virus. The second withdrawal resulted in chronic hepatitis B with HBeAg positivity from OBI. As a result, improper medication withdrawal may result in disease progression. We also speculated whether long-term untreated OBI with positive HBeAg would more likely to result in the occurrence of adverse consequences such as liver cirrhosis and HCC, which has been reported in OBI with HBeAg negativity (Perisetti et al., 2021; Huang et al., 2022).

Whether HBV reactivation was caused by latent virus replication or a new HBV subtype infection is unknown. Given the coexistence of wild (G1896) and mutant (A1896) types of pre-C in the patient in August 2021, it is reasonable to assume that HBsAg was caused by the reactivation of the latent virus, which was now dominated by wild-type virus. Previous studies using nucleotide analog (NA) therapy demonstrated that the wild-type virus could replace pre-Core/BCP mutants during antiviral therapy (Cho et al., 2000; Lau et al., 2020). During the follow-up period, the patient may evolve from the coexistence of wild and mutant sequences to the sole wild-type virus. Assume, however, that the patient continues to discontinue antiviral therapy with increased mutant strains. In that case, subsequent follow-up visits may reveal negative HBeAg, which may not improve the disease. Furthermore, because the S gene’s open reading frame partially overlaps with that of the reverse transcriptase (RT) gene, mutations in the S gene cause antiviral resistance mutations to appear in the RT domain. The variation of S gene not only Strongly Affect HBsAg Detection (Kuhns et al., 2021; Wang et al., 2021), but also leads to HBV drug resistance and immune escape (Delfino et al., 2021). Drug resistance must be considered when a patient experiences a poor curative effect during a follow-up visit.

Finally, in our case, antiviral therapy effectively inhibited viral replication in OBI with positive HBeAg and detectable serum HBV DNA. Nonetheless, abrupt drug discontinuation can hasten the progression of the disease. It is still debatable whether OBI requires antiviral therapy. Long-term observation of large cohorts is needed to elucidate the potential impact of antiviral treatment on OBI. We recommend NA antiviral therapy for OBI patients with positive HBeAg and high serum HBV DNA, especially for patients with a family history of chronic hepatitis B and/or HCC. Meanwhile, detection of HBV gene variation and drug resistance genes should be strengthened both before and during treatment to optimize antiviral therapy and efficacy. Strengthening patient compliance education is helpful to guarantee the curative effect of antiviral therapy.

## Data Availability Statement

The original contributions presented in the study are included in the article/supplementary material, further inquiries can be directed to the corresponding author.

## Ethics Statement

Ethical review and approval was not required for the study on human participants in accordance with the local legislation and institutional requirements. Written informed consent for participation was not required for this study in accordance with the national legislation and the institutional requirements.

## Author Contributions

All authors listed have made a substantial, direct, and intellectual contribution to the work and approved it for publication.

## Funding

This work was supported by a grant from the Medical Science and Technology Fund of Chongqing Municipal Public Health Bureau (Grant No. 2015ZDXM008).

## Conflict of Interest

The authors declare that the research was conducted in the absence of any commercial or financial relationships that could be construed as a potential conflict of interest.

## Publisher’s Note

All claims expressed in this article are solely those of the authors and do not necessarily represent those of their affiliated organizations, or those of the publisher, the editors and the reviewers. Any product that may be evaluated in this article, or claim that may be made by its manufacturer, is not guaranteed or endorsed by the publisher.
